# The complete chloroplast genome sequence of *Strobilanthes tonkinensis* Lindau

**DOI:** 10.1080/23802359.2021.1934144

**Published:** 2021-05-27

**Authors:** Liu Fang, Ying Wang, Cui-Ling Guo, Xing-Ya Wang

**Affiliations:** College of Pharmaceutical Sciences, Zhejiang Chinese Medical University, Hangzhou, China

**Keywords:** *S. tonkinensis* Lindau, Acanthaceae, phylogenomics

## Abstract

*Strobilanthes tonkinensis* Lindau is a member of the family Acanthaceae, which was originated from Yunnan province of China and is used as tea and health promotion. Here, we reported the complete chloroplast genome sequence of *S. tonkinensis* using Illumina high-throughput sequencing approach. The size of the chloroplast genome is 144,765 bp in length, containing a pair of inverted repeats (IRs, 17,362 bp) that are separated by the large single-copy (LSC, 92,248 bp), and small single-copy (SSC, 17,793 bp) regions. A total of 129 genes were identified, including 37 tRNA genes, 8 rRNA genes, and 84 protein-coding genes. The overall GC content is 38.21%. Phylogenetic analysis indicated that *S. tonkinensis* is closely related to *Strobilanthes cusia* and *Strobilanthes bantonensis*.

*Strobilanthes tonkinensis* Lindau, 1897, also called *Semnostachya menglaensis* (Wu et al. 2011), is a rare plant of the genus Strobilanthes and a perennial herb of the family Acanthaceae, mainly distributed in Yunnan, China (Srikun 2017). *S. tonkinensis* has been widely used as natural herbal tea and spices in the Southern part of China, which has a distinctive fragrance similar to glutinous rice (Wu et al. 2011; Zhang et al. 2015). In addition, *S. tonkinensis* contains a large amount of squalene, which may be responsible for its biological function, such as antioxidative activity (Yang et al. 2014; Srikun 2017). The complete chloroplast (cp) genome has been widely used as a valuable source of data for understanding evolutionary biology due to its conservation in gene content (Sun et al. 2020). In this study, we sequenced and assembled the cp genome of *S. tonkinensis* for the first time to provide necessary genomic resources for further study and provide important information for the phylogeny of Acanthaceae.

Fresh leaves of *S. tonkinensis* were collected from Yunnan, China (100°43′51.3804″E, 21°40′55.632 N). The voucher specimen and DNA sample were deposited at the Herbarium of College of pharmaceutical sciences, Zhejiang Chinese Medical University (X. Wang, xywang@zcmu.edu.cn) under the voucher number ZCMU4C505. The total genomic DNA was extracted from *S. tonkinensis* leaves by a modified CTAB method (Doyle and Doyle 1987). After DNA extraction, the genomic DNA was segmented by ultrasound, and a sequencing library with an insert size of 320 bp fragments was constructed by PCR amplification. Then the genomic library was sequenced using the Illumina Novaseq 6000 platform (Illumina, San Diego, CA) in Genepioneer Biotechnologies (Nanjing, China) and approximately 9.11 GB of clean data were obtained. The filtered data were then assembled using NOVOPlasty software (Dierckxsens et al. 2016), with *Strobilanthes cusia* as the reference (GenBank accession number: MG874806.1). Then the assembled sequences of *S. tonkinensis* were annotated using CpGAVAS (Liu et al. 2012) and checked with DOGMA and BLAST (Wyman et al. 2004).

The complete chloroplast genome of *S. tonkinensis* (GenBank accession number: MW525447) is 144,765 bp in length with a typical quadripartite structure containing a pair of inverted repeats (IRs, 17,362 bp), a large single-copy region (LSC, 92,248 bp), and a small single-copy region (SSC, 17,793 bp). The total GC content of the whole genome, IRs, LSC, and SSC were 38.21%, 36.53%, 32.49%, and 45.61%, respectively. A total of 129 genes were successfully annotated, including 8 rRNA genes, 37 tRNA genes, and 84 protein-coding genes. Of which, 16 genes contain one intron, 2 genes contain two introns, and 8 genes were duplicated in the IRs region.

In order to explore the phylogenetic position and evolutionary relationship of *S. tonkinensis*, a phylogenetic analysis was conducted between *S. tonkinensis* and other 23 complete chloroplast genomes of the Acanthaceae downloaded from the NCBI Nucleotide Resource database, *Ginkgo biloba* was used as the outgroup. MAFFT version 7 software (Katoh et al. 2005) was used to align the above sequences. A maximum likelihood (ML) tree was built using RA × ML (Alexandros 2014) with 1000 bootstrap and with GTRGAMMA as the best nucleotide substitution model. The results indicated that *S. tonkinensis* belongs to the family Acanthaceae and is closely related to *Strobilanthes cusia* and *Strobilanthes bantonensis* (Figure 1).

**Figure 1.**
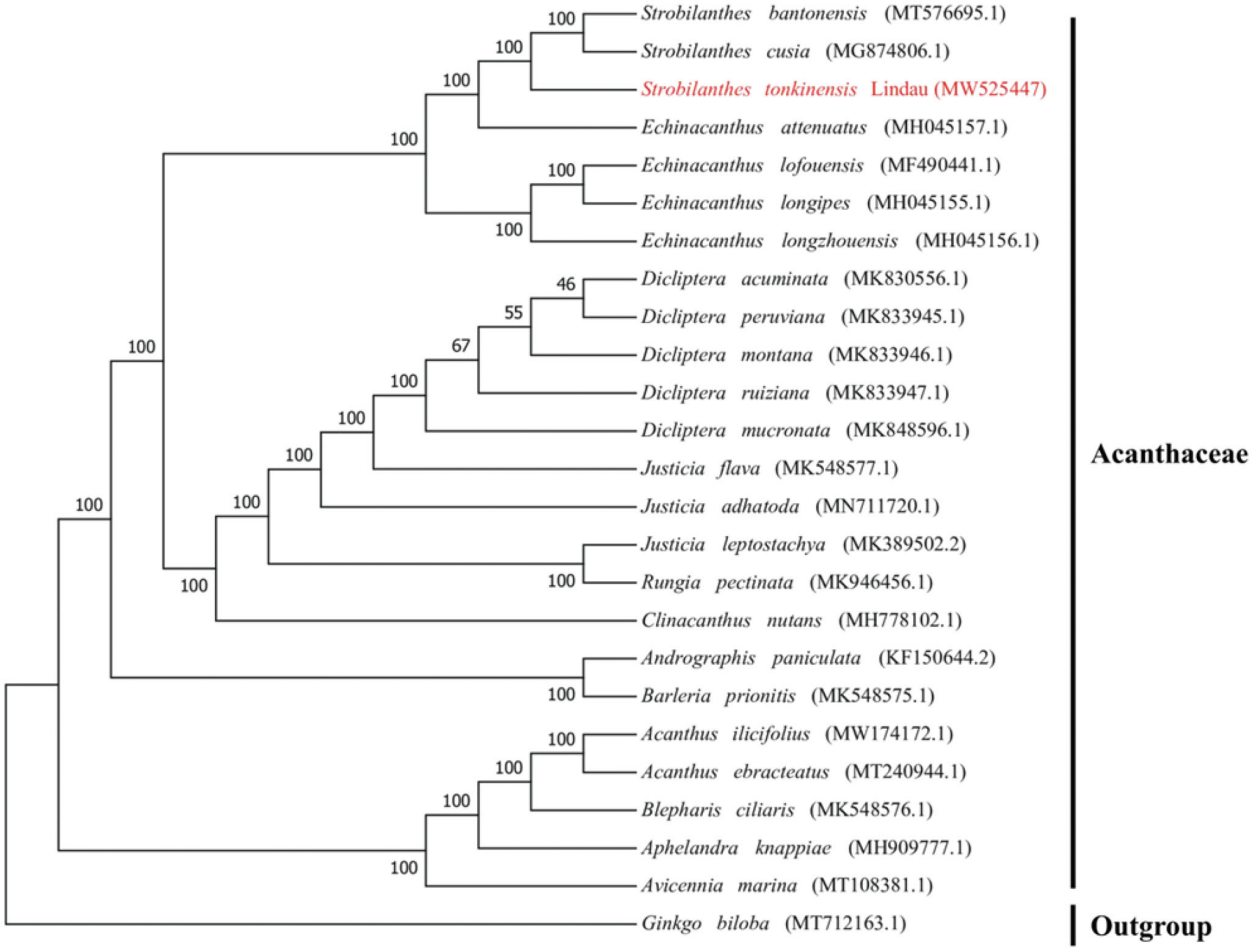
Maximum likelihood phylogenetic tree based on 25 complete chloroplast genomes. The bootstrap values are list on the nodes.

## Data Availability

The genome sequence data that support the findings of this study are openly available in GenBank of NCBI at https://www.ncbi.nlm.nih.gov, under the accession number MW525447. The associated BioProject, SRA, and Bio-Sample numbers are PRJNA727488, SRR14429881, and SAMN19022189, respectively.
